# Docosahexaenoic acid attenuates the early inflammatory response following spinal cord injury in mice: *in-vivo* and *in-vitro* studies

**DOI:** 10.1186/1742-2094-11-6

**Published:** 2014-01-10

**Authors:** Irene Paterniti, Daniela Impellizzeri, Rosanna Di Paola, Emanuela Esposito, Stacy Gladman, Ping Yip, John V Priestley, Adina T Michael-Titus, Salvatore Cuzzocrea

**Affiliations:** 1 Department of Biological and Environmental Sciences, University of Messina, Viale Ferdinando Stagno D’Alcontres, 98166 Messina, Italy; 2 Barts and The London School of Medicine and Dentistry, Queen Mary University of London, Whitechapel, London, UK

**Keywords:** DHA, Inflammation, Omega-3, Oxidative stress, Spinal cord injury

## Abstract

**Background:**

Two families of polyunsaturated fatty acid (PUFA), omega-3 (ω-3) and omega-6 (ω-6), are required for physiological functions. The long chain ω-3 PUFAs, eicosapentaenoic acid (EPA) and docosahexaenoic acid (DHA), have significant biological effects. In particular, DHA is a major component of cell membranes in the brain. It is also involved in neurotransmission. Spinal cord injury (SCI) is a highly devastating pathology that can lead to catastrophic dysfunction, with a significant reduction in the quality of life. Previous studies have shown that EPA and DHA can exert neuroprotective effects in SCI in mice and rats. The aim of this study was to analyze the mechanism of action of ω-3 PUFAs, such as DHA, in a mouse model of SCI, with a focus on the early pathophysiological processes.

**Methods:**

In this study, SCI was induced in mice by the application of an aneurysm clip onto the dura mater via a four-level T5 to T8 laminectomy. Thirty minutes after compression, animals received a tail vein injection of DHA at a dose of 250 nmol/kg. All animals were killed at 24 h after SCI, to evaluate various parameters implicated in the spread of the injury.

**Results:**

Our results in this *in-vivo* study clearly demonstrate that DHA treatment reduces key factors associated with spinal cord trauma. Treatment with DHA significantly reduced: (1) the degree of spinal cord inflammation and tissue injury, (2) pro-inflammatory cytokine expression (TNF-α), (3) nitrotyrosine formation, (4) glial fibrillary acidic protein (GFAP) expression, and (5) apoptosis (Fas-L, Bax, and Bcl-2 expression). Moreover, DHA significantly improved the recovery of limb function.

Furthermore, in this study we evaluated the effect of oxidative stress on dorsal root ganglion (DRG) cells using a well-characterized *in-vitro* model. Treatment with DHA ameliorated the effects of oxidative stress on neurite length and branching.

**Conclusions:**

Our results, *in vivo* and *in vitro*, clearly demonstrate that DHA treatment reduces the development of inflammation and tissue injury associated with spinal cord trauma.

## Background

Spinal cord injury (SCI) is defined as an acute traumatic lesion of neural elements in the spinal canal (spinal cord and cauda equina), resulting in a change, either temporary or permanent, in normal motor, sensory, or autonomic function.

It is estimated that each year approximately 130,000 individuals worldwide sustain an acute SCI, joining a recently estimated 2,500,000 who are already living with chronic paralysis [1,2]. Spinal cord injury triggers the activation of the innate as well as the adaptive immune system. Innate immune cells present in the central nervous system (CNS, microglia or astrocytes) or derived from the blood (monocytes, macrophages and polymorphonuclear leukocytes, such as neutrophils) respond very quickly to the injury [3,4], but their response is nonspecific, since the molecules produced (reactive oxygen species, such as superoxide) cause collateral damage to surrounding tissue. Whether activation of the immune system is detrimental or beneficial after SCI is a question that is not easy to answer [5]. The beneficial or detrimental effect of neuroinflammation probably depends on the inflammatory mediators released during particular phases of the inflammation that follows SCI [6]. Spinal cord injury usually begins with a sudden, traumatic blow to the spine that causes local segmental damage to the spinal cord, which is called primary injury [7,8]. The primary damage to tissue is followed by a second phase of tissue degeneration, the secondary injury that can occur over weeks or even months [8]. In secondary injury, acute inflammation can develop into a chronic process if feedback mechanisms fail to inhibit amplification of the inflammatory response. Chronic inflammation leads to a continuous influx of neutrophils, macrophages, lymphocytes, and eosinophils from the circulation, causing more destruction and scarring of tissue. Cell death resulting from all of these mechanisms occurs through necrotic and apoptotic phenomena or autophagia. In addition to the spinal cord, spinal nerves and their associated dorsal root ganglion (DRG) cells can be subject to mechanical deformation and hypoxia associated with pathology, such as spinal stenosis and spinal trauma. However, there is very limited information on the response of adult DRG neurons to such stressors, despite their obvious importance to the function of the spinal cord.

There are no effective treatments for SCI, making this condition one of the most challenging research topics. Major progress has been made in preclinical studies on neuroprotection and regeneration [2,9], but this progress has not yet been translated to the clinic. Over the past 10 years, there has been increased interest in the health benefits of polyunsaturated fatty acids (PUFAs), with evidence emerging that ω-3 PUFAs have significant therapeutic potential in a variety of CNS disorders, including Zellweger syndrome, schizophrenia, depression, and Alzheimer’s disease [10,11]. PUFAs are structural components of phospholipids, which are the main constituents of cell membranes. The PUFAs of the ω-3 series include α-linolenic acid, eicosapentaenoic acid (EPA) and docosahexaenoic acid (DHA), the latter two being the longer chain compounds, whereas α-linolenic acid is the biosynthetic precursor. Recent evidence shows that ω-3 PUFAs can modulate several of the processes that contribute to secondary degeneration in the CNS [12]. There is evidence that ω-3 PUFAs have antioxidant effects as well as anti-inflammatory effects through inhibition of the production of pro-inflammatory cytokines. PUFAs can also block apoptosis, and several studies have documented their neuroprotective effects *in vitro* and *in vivo*. Recently, several studies have demonstrated that ω-3 PUFAs could have significant therapeutic potential in the treatment of SCI [13-17]. In these studies, the fatty acids were administered within the first hour after injury and the outcome was characterized using behavioral tests and tissue analysis several weeks after injury. Thus, there is still limited information on the modulation by DHA of the very early events after injury.

The purpose of this study is to investigate the effects of ω-3 PUFAs treatment in a mouse compression model of SCI and, in particular, to explore whether DHA could reduce the early inflammatory response induced after SCI. This experimental model of SCI express some of the features of the injury response that are seen in human beings and are routinely used in research to assess pain pathways, biochemical markers, morphology, and path physiology. To improve SCI management, it is of vital importance to explore novel therapeutics and gain understanding of the pathophysiological processes that follow traumatic injury to the spinal cord. In addition, in this study we aim to provide information on the response of adult DRG neurons to oxidative stress, determining whether DHA is neuroprotective in a well-characterized *in-vitro* model of injury, using adult mouse DRG neurons.

## Methods

### *In-vivo* procedures

#### ***Animals***

Male adult CD1 mice (25 to 30 g, Harlan Nossan, Milan, Italy) were housed in a controlled environment and provided with standard rodent chow and water. Mice were housed in stainless steel cages in a room kept at 22 ± 1°C with a 12-h light, 12-h dark cycle. The animals were acclimatized to their environment for 1 week and had *ad-libitum* access to tap water and rodent standard diet. The study was approved by the University of Messina Review Board for the care of animals. All animal experiments complied with regulations in Italy (DM 116192) as well as EU regulations (OJ of EC L 358/1 12/18/1986).

#### ***SCI***

Mice were anaesthetized using chloral hydrate (400 mg/kg body weight). A longitudinal incision was made on the midline of the back, exposing the paravertebral muscles. These muscles were dissected away exposing the T5 to T8 vertebrae. The spinal cord was exposed via laminectomy and SCI was produced by extradural compression of the spinal cord using an aneurysm clip (using the aneurysm clip applicator oriented in the bilateral direction) with a closing force of 24 g, at the T6 to T7 level, as described by Rivlin and Tator [18]. In all injured groups, the spinal cord was compressed for 1 min. Sham-operated animals were only subjected to laminectomy. Following surgery, 1.0 ml of normal saline was administered subcutaneously, to replace the blood volume lost during the surgery. During their recovery from anesthesia, the mice were placed on a warm heating pad and covered with a warm towel. During the period following injury, the animals’ bladders were manually voided twice a day until the mice were able to regain normal bladder function. Spinal cord tissues were collected at 24 hours following trauma.

##### 

**Experimental groups** Mice were randomly allocated into the following groups:

1) SCI + vehicle group. Mice were subjected to SCI plus administration of saline (via tail vein injection), 30 min after SCI (N = 40);

2) DHA group. As for the SCI + vehicle group, except that DHA (250 nmol/kg, as used in a previous study [12], was injected intravenously (tail vein injection), 30 min after SCI (*N* = 40).

3) Sham + vehicle group. Mice were subjected to the same surgical procedures as the above groups except that the aneurysm clip was not applied and saline was injected intravenously 30 min after SCI (*N* = 40);

4) SCI PPARα KO + vehicle group. Mice were subjected to SCI plus administration of saline (via tail vein injection), 30 min after SCI (*N* = 40);

5) SCI PPARα KO + DHA group. As for the SCI + vehicle group except that DHA (250 nmol/kg, as used in a previous study [12] was injected intravenously (tail vein injection), 30 min after SCI (*N* = 40);

6) Sham PPARαKO + vehicle group. Mice were subjected to the same surgical procedures as the above groups except that the aneurysm clip was not applied and saline was injected intravenously 30 min after SCI (*N* = 40);

Mice (*N* = 10 from each group for each parameter) were sacrificed at 24 h after SCI to evaluate the various parameters.

In a separate set of experiments, another ten animals for each group, both wild-type (WT) mice and PPARα KO mice, were observed until 10 days after SCI, to evaluate the motor score. The animals of the DHA group received treatment with DHA (250 nmol/kg, as tail vein injection) 30 min after SCI and daily until day 9; the motor score was assessed daily.

### Preparation of the solution of docosahexaenoic acid (DHA)

The fatty acid solution for intravenous administration was prepared as follows: 1 M stock solution of DHA (Sigma, UK) was prepared in ethanol under nitrogen and then stored at -20°C. For the experiment, 5 μl aliquots of DHA (1 M in ethanol) were taken and diluted in sterile NaCl 0.9% and the pH adjusted to 7.4. The DHA solution (250 nmol/kg body weight), was administered 30 minutes after injury in a tail vein, under light anesthesia using sevoflurane.

### Grading of motor disturbance

The motor function of mice subjected to compression trauma was assessed once a day for 10 days after injury. Recovery from motor impairment was graded using the Basso mouse scale (BMS) open-field score [19], since the BMS has been shown to be a valid locomotor rating scale for mice. The evaluations were made by two blind observers for all analyzed groups. The BMS scale ranges from 0 (indicating complete paralysis) to 9 (indicating normal hind limb function), and rates locomotion on such aspects of hind limb function as weight support, stepping ability, coordination, and toe clearance. The BMS score was determined for ten mice in each group.

### Immunohistochemical localization of TNF-α, nitrotyrosine, iNOS, Fas-L, GFAP, Bax, and Bcl-2

Spinal cord tissues were taken at 24 h following trauma, and were fixed for 24 h in paraformaldehyde solution (4% in PBS 0.1 M) at room temperature, dehydrated by graded ethanol, and embedded in Paraplast (Sherwood Medical, Mahwah, NJ). Sections 8 μm thick were cut from the paraffin-embedded tissue. After deparaffinization, endogenous peroxidase was quenched with 0.3% (v/v) hydrogen peroxide in 60% (v/v) methanol for 30 minutes. The sections were permeabilized with 0.1% (w/v) Triton X-100 in PBS for 20 minutes. Nonspecific adsorption was minimized by incubating the section in 2% (v/v) normal goat serum in PBS for 20 minutes. Endogenous biotin or avidin binding sites were blocked by sequential incubation for 15 minutes with biotin and avidin (Vector Laboratories, Burlingame, CA, USA), respectively. Sections were incubated overnight with anti-nitrotyrosine rabbit polyclonal antibody (SantaCruz Biotechnology 1:500 in PBS, v/v), anti-iNOS rabbit polyclonal antibody (BD Transduction 1:500 in PBS, v/v), anti-Bax rabbit polyclonal antibody (SantaCruz Biotechnology 1:500 in PBS, v/v), or anti-Bcl-2 rabbit polyclonal antibody (SantaCruz Biotechnology 1:500 in PBS, v/v), anti-Fas ligand (Fas-L) rabbit polyclonal antibody (SantaCruz Biotechnology 1:500 in PBS, v/v), anti-tumor necrosis factor (TNF)-α rabbit polyclonal antibody (SantaCruz Biotechnology 1:500 in PBS, v/v), and with anti-glial fibrillary acidic protein (anti-GFAP) mouse monoclonal antibody (1:500; Cell Signaling Technology). Sections were washed with PBS and incubated with peroxidase-conjugated bovine anti-mouse immunoglobulin G (IgG) secondary antibody or peroxidase-conjugated goat anti-rabbit IgG (1:2,000 Jackson Immuno Research, West Grove, PA, USA). Specific labeling was detected with a biotin-conjugated goat anti-rabbit IgG or biotin-conjugated goat anti-mouse IgG and avidin-biotin peroxidase complex (Vector Laboratories, Burlingame, CA, USA). To verify the binding specificity for nitrotyrosine, iNOS, Bax, and Bcl-2, Fas-L, TNF-α, and anti-GFAP, control sections were also incubated with only the primary antibody (no secondary) or with only the secondary antibody (no primary). In these controls, no positive staining was found in the sections, indicating that the immunoreaction was positive in all the experiments. Immunohistochemical photographs were assessed by densitometric analysis using an imaging densitometer (AxioVision, Zeiss, Milan, Italy). Briefly, for each tissue section at least ten optical fields in the area at the boundary between the necrotic core and the penumbra area (perilesioned area) and ten optical fields distal to the damaged area were counted, as described previously [20]. Replicates for each experimental condition and histochemical staining were obtained from each mouse in each experimental group. In sham-operated mice, the central areas of corresponding tissue sections were taken as reference points, and a comparable number of optical fields were counted. Data are expressed as a percentage of total tissue area, as described previously [21].

### H & E staining

Paraffin tissue sections (thickness, 5 μm) were deparaffinized with xylene, stained with H & E, and studied using light microscopy (Dialux 22, Leitz, Germany). Segments of each spinal cord were evaluated by an experienced histopathologist. Damaged neurons were counted and the histopathological changes of the gray matter were scored on a 6-point scale [22]: 0 = no lesion observed; 1 = gray matter contained one to five eosinophilic neurons; 2 = gray matter contained five to ten eosinophilic neurons; 3 = gray matter contained more than ten eosinophilic neurons; 4 = small infarction (less than one-third of the gray matter area); 5 = moderate infarction; (one-third to one-half of the gray matter area); 6 = large infarction (more than one-half of the gray matter area). The scores from all the sections from each spinal cord were averaged to give a final score for an individual mouse. All the histological studies were performed in a blinded fashion.

### Preparation of cytosolic and nuclear extracts from spinal cord and Western blot analysis

Cytosolic and nuclear extracts were prepared as described previously [23], with slight modifications. Briefly, 2 cm tissue segments containing the lesion from each mouse were suspended in extraction buffer A containing 0.2 mM phenylmethylsulfonyl fluoride (PMSF), 0.15 mM pepstatin A, 20 mM leupeptin, 1 mM sodium orthovanadate, homogenized at the highest setting for 2 minutes, and centrifuged for 15 minutes at 4°C. Supernatants represented the cytosolic fraction. The pellets (containing enriched nuclei) were resuspended in buffer B containing 1% Triton X-100, 150 mM NaCl, 10 mM Tris-HCl pH 7.4, 1 mM ethylene glycol tetraacetic acid, 1 mM ethylenediaminetetraacetic acid, 0.2 mM PMSF, 20 μm leupeptin, 0.2 mM sodium orthovanadate. After centrifugation for 30 minutes at 15,000 *g* at 4°C, the supernatants containing the nuclear protein were stored at -80°C for further analysis. The levels of IκB-α, Bax, and Bcl-2 were quantified in the cytosolic fraction from spinal cord tissue collected after 24 h after SCI, whereas NF-κB p65 levels were quantified in the nuclear fraction. Protein concentration was determined with the Bio-Rad protein assay kit. Proteins (40 μg) were dissolved in Laemmli Sample Buffer, boiled for 5 minutes, and subjected to sodium dodecyl sulfate PAGE (10% or 12% polyacrylamide). Proteins were separated electrophoretically and transferred to nitrocellulose membranes. Membranes were blocked with 5% (w/v) nonfat dried milk in buffered saline (PM) for 45 minutes at room temperature and subsequently probed with specific antibodies: anti-IκB-α (1:1.000; Santa Cruz Biotechnology), anti-Bax (1:500; Santa Cruz Biotechnology), anti-Bcl-2 (1:500; SantaCruz Biotechnology), anti-Fas-L (1:500; SantaCruz Biotechnology), anti-iNOS (1:1000; BD transduction), or anti- NF-κB p65 (1:500 SantaCruz Biotechnology) in 1× PBS, 5% w/v nonfat dried milk, and 0.1% Tween-20 (PMT) at 4°C overnight. Membranes were incubated with peroxidase-conjugated bovine anti-mouse immunoglobulin G (IgG) secondary antibody or peroxidase-conjugated goat anti-rabbit IgG (1:2.000, Jackson Immuno Research, West Grove, PA) for 1 h at room temperature.

To ascertain that Western blots were loaded with equal amounts of protein lysates, they were also incubated in the presence of the antibody against β-actin (for cytosolic extract) or lamin A/C (for nuclear extract) proteins (1:10.000; Sigma-Aldrich Co., Milan, Italy). Signals were detected with the enhanced chemiluminescence detection system reagent, according to the manufacturer’s instructions (SuperSignal West Pico Chemiluminescent Substrate, Pierce, Thermo Fisher Scientific Inc., Rockford, IL, USA).

### Immunofluorescence

After deparaffinization and rehydration, detection of GFAP, TNFα, Bax, and Iba-1 was carried out after boiling in 0.1 M citrate buffer for 1 min. Nonspecific adsorption was minimized by incubating the section in 2% (v/v) normal goat serum in PBS for 20 min. Sections were incubated with mouse monoclonal anti-GFAP (1:100, v/v Santa Cruz Biotechnology), polyclonal rabbit anti-TNFα (1:100, v/v Santa Cruz, Biotechnology), rabbit anti-Bax (1:100, v/v Santa Cruz Biotechnology), or mouse monoclonal anti-Iba-1 (1:100, v/v Santa Cruz Biotechnology) antibody in a humidified chamber for O/N at 37°C. Sections were washed with PBS and incubated with secondary antibody FITC-conjugated anti-mouse Alexa Fluor-488 antibody (1:2000 v/v Molecular Probes, UK) and with TEXAS RED-conjugated anti-rabbit Alexa Fluor-594 antibody (1:1000 in PBS, v/v Molecular Probes, UK) for 1 h at 37°C. Sections were washed; for nuclear staining 2 μg/ml 4′,6′-diamidino-2-phenylindole (Hoechst, Frankfurt; Germany) in PBS was added. Cells were observed at ×20 magnification using a Leica DMRD microscope (Leica). All images were digitalized at a resolution of 8 bits into an array of 2048 × 2048 pixels. Optical sections of fluorescence specimens were obtained using a HeNe laser (543 nm), an UV laser (361 to 365 nm) and an Ar laser (458 nm) at a 1-min, 2-s scanning speed with up to eight averages; 1.5-μm sections were obtained using a pinhole of 250 nm. Contrast and brightness were established by examining the most brightly labeled pixels and applying settings that allowed clear visualization of structural details while keeping the highest pixel intensities close to 200. The same settings were used for all images obtained from the other samples that had been processed in parallel. Digital images were cropped and figure montages prepared using Adobe Photoshop 7.0 (Adobe Systems; Palo Alto, CA).

### Materials

Unless otherwise stated, all compounds were obtained from Sigma-Aldrich Company Ltd (Milan, Italy). All stock solutions were prepared in nonpyrogenic saline (0.9% NaCl; Baxter, Italy) or 10% dimethyl sulfoxide.

### Statistical evaluation

All values in the figures and the text are expressed as mean ± standard error of the mean (SEM). Results shown in the figures are representative of at least three experiments performed on different *in-vivo* experimental days. In each experiment, we used five animals per group, unless otherwise indicated. The results were analyzed by one-way analysis of variance followed by a Bonferroni post-hoc test for multiple comparisons. A *P* value of less than 0.05 was considered significant.

### *In-vitro* procedures

#### ***Primary DRG neuron culture***

Primary neuronal cultures were prepared according to the method of Gavazzi and colleagues [24], with some modifications, as described here. Adult male CD1 mice 25 to 30 g (Charles Rivers, UK) were sacrificed by inhalation of CO_2_, followed by decapitation. All procedures were carried out in accordance with the UK Animals (Scientific Procedures) Act 1986 and associated guidelines. Cervical to lumbar level DRGs were removed and cleaned before being dissociated chemically with 0.125% collagenase (Type 4; Worthington, UK) at 37°C for 45 minutes. The collagenase concentration was then raised to 0.25% for another 45 minutes and the ganglia were washed and dissociated with 0.25% trypsin (Sigma, UK) for a further 15 minutes. The tissue was then triturated with a pipette tip until homogeneously dissociated in serum-free medium (BSF-2) consisting of 1% N-2 supplement (Invitrogen, UK), 0.3% bovine serum albumin (BSA) (Fraction V; Sigma, UK) and 100 unit/ml penicillin and 100 μg/ml streptomycin (Sigma, UK) in Ham’s F-12 medium (Invitrogen, UK). The cell suspension was then centrifuged at 400 rpm (30 *g*) for 5 minutes followed by resuspension and a second centrifugation through a 15% BSA (Sigma, UK) cushion at 900 rpm (153 *g*) for 10 minutes. Cellular debris collected at the BSA/BSF-2 interface was removed along with the remaining supernatant. The pelleted cells were resuspended in BSF-2 containing glial cell-line derived neurotrophic factor (25 ng/ml, R&D Systems, UK). The cell suspension was plated out onto either glass coverslips double-coated with poly-D-lysine (5 μg/ml; Sigma, UK) and laminin (10 μg/ml; Sigma, UK) and left to adhere for 1 h before BSF-2 was added to cover the surface of the wells. Cultures were maintained at 37°C in a humidified atmosphere with 5% CO_2_ / 95% air for 48 h prior to further experiments.

### Preparation of omega-3 PUFAs

Stock solutions of 1 M DHA (Sigma, UK) were diluted to 1 mM with BSF-2 under nitrogen and stored at -20°C. These stock solutions were then diluted as required with BSF-2 and adjusted to pH 7.4 using sodium hydroxide. The chosen final concentration of DHA was 1 μM. Ethanol was added to the medium and used as the vehicle control in the *in-vitro* experiments, as this was the solvent initially used to dissolve the PUFA. The final concentrations of ethanol in the vehicle-exposed cultures were identical to those present in the DHA preparations.

### Oxidative stress injury model

A single 12-well culture plate (Sigma, UK) was used for each experiment. Existing BSF-2 was first removed from all wells in culture plates and fresh BSF-2 was added. Dorsal root ganglias were cultured for 48 h before 1 μM DHA final concentration or vehicle was added, immediately before the induction of oxidative stress with hydrogen peroxide (Sigma, UK) 10 nM for 24 hours. For dilution of stock solutions of both DHA and H_2_O_2,_ BSF-2 media was used.

### Immunocytochemistry

After 24 h from the induction of the oxidative stress, DRG cultures were fixed with ice-cold 4% paraformaldehyde for 5 minutes at room temperature. Cultures were stained with anti-β-tubulin III primary antibody (1:1000 Sigma, UK). Cultures were then washed and incubated with the appropriate anti-mouse secondary antibodies with Alexa Fluor-488 (1:2000 Molecular Probes, UK). Cultures were also counterstained with the fluorescent nuclear stain, 4′,6′-diamidino-2-phenylindole (Hoechst 2 μg/ml; Sigma, UK). Cells were observed at ×20 magnification using a Leica DMRD microscope (Leica, UK). Images were captured using a Hamamatsu (Hamamatsu, Japan) digital camera and HiPic32 imaging software. Adobe Photoshop 7 was used to produce micrographs.

### Neurite outgrowth assay

Neurite growth was assessed for the entire neuronal population. Cultures were stained with β-tubulin III for visualization of neurites. For each experimental condition, images of 50 neurons were recorded for each animal (*n* = 3) and imported into NeuronJ, an ImageJ plugin (National Institute of Health, USA).

The length of the longest neurite, total neurite outgrowth, and number of branches was also measured; images of 50 neurons were recorded for each animal (*n* = 3) and imported into the MetaMorph analysis program (Molecular Devices, USA). The number of neurons with neurites was also recorded.

### Statistical analysis

For the *in-vitro* study, results are expressed as mean ± SEM of three experiments, with each experiment using ganglia pooled from two animals. A two-way analysis of variance (ANOVA) was used to compare the overall effect of treatment and injury on cell death, followed by post-hoc multiple pairwise comparisons using Tukey’s test, with *P* < 0.05 indicating a statistically significant difference.

## Results

### *In-vivo* study

#### ***DHA reduces the severity of SCI in the thoracic region of spinal cord***

**Figure 1:**
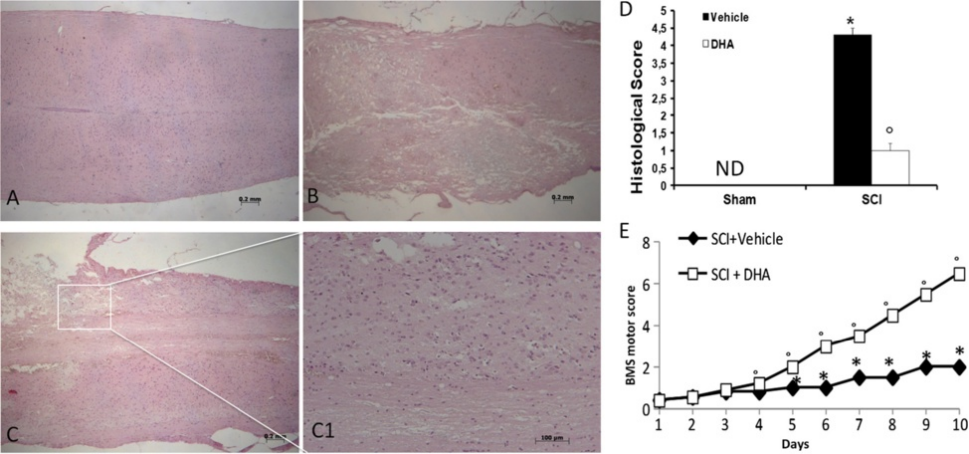
**Effect of DHA treatment on histological alterations of the spinal cord tissue 24 h after injury and on hind limb motor disturbance.** No histological alterations have been found in the spinal cord tissue collected from sham-operated mice (**A**, see histological score **D**). A significant damage to the spinal cord was assessed in SCI-operated mice stained with H & E (**B** see histological score **D**). The treatment with DHA resulted in a significant decrease in the extent and severity of the histological signs (**C**, see histological score **D**). Moreover, the degree of motor disturbance was assessed every day until 10 days after SCI by Basso mouse scale (BMS) open-field score. Treatment with DHA administered 30 min after trauma reduces the motor disturbance after SCI **(E)**. This figure is representative of at least three experiments performed on different experimental days. ND: not detectable. Values shown are mean ± SEM of ten mice for each group; *P < 0.01 vs sham; °*P* < 0.01 vs SCI.

The severity of trauma at the level of the perilesional area, assessed by the presence of edema as well as assessment of alterations of white matter, was evaluated by H & E staining (Figure 1) at 24 h after trauma. Significant damage to the spinal cord was observed in tissue from mice subjected to SCI (Figure 1B, see histological score Figure 1D) when compared with sham-operated mice (Figure 1A, see histological score Figure 1D). Notably, a significant protection against SCI was observed in DHA-treated mice (Figure 1C,C1, see histological score Figure 1D). To evaluate whether histological damage to the spinal cord was associated with a loss of motor function, the BMS open-field score was used. Motor function was not impaired in sham mice. Mice subjected to SCI showed significant deficits in hind limb movement (Figure 1E) starting with the first evaluation performed 24 h after trauma. In the DHA-treated mice group, the neurological score improved in a statistically significant way beginning at four days after chronic administration, compared with the SCI-vehicle mice group, and persisted up to 10 days after SCI.

### Effect of DHA on IκB-a degradation and NF-κB p65 nuclear localization

**Figure 2:**
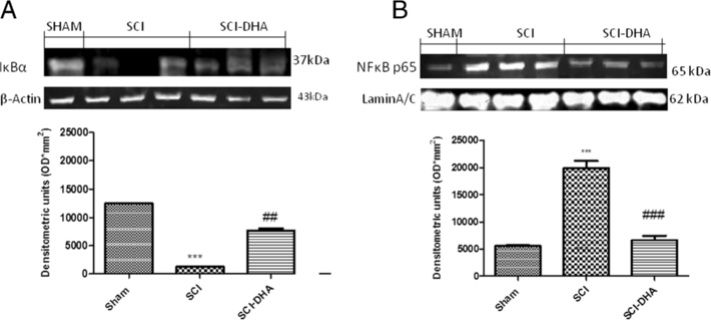
**Effects of DHA treatment on IκBα and nuclear NF-κB p65.** By Western blot analysis, a basal level of IκB-α was detected in the spinal cord from sham-operated animals **(A)**, whereas IκB-α levels were substantially reduced in SCI mice **(A)**. DHA treatment reduced the SCI-induced IκB-α degradation **(A)**. In addition, SCI caused a significant increase in nuclear NF-κB p65 compared with sham-operated mice **(B)**. DHA treatment significantly reduced the phosphorylation of p65 on Ser536 and the translocation into the nucleus of NF-κB p65 **(B)**. A representative blot of lysates obtained from each group is shown, and densitometry analysis of all animals is reported. Respective densitometry analysis of protein bands from three separated experiments is reported (***P < 0.001 vs. Sham; ^###^*P* < 0.005 vs. SCI; ## *P*<0.05 vs.SCI).

To analyze the molecular mechanisms by which DHA treatment may attenuate the development of SCI, we evaluated by Western blot analysis the classical NF-κB pathway, evaluating both IκB-α levels and NF-κB nuclear localization. A basal level of IκB-α was detected in the spinal cord sections from sham-operated animals (Figure 2A), whereas SCI substantially reduced IκB-α levels (Figure 2A). DHA prevented the SCI-induced IκB-α degradation and restored IκB-α to levels similar to those in sham-operated mice (Figure 2A). In addition, SCI caused a significant increase in the nuclear NF-κB p65 levels compared with the sham-operated mice (Figure 2B). DHA treatment significantly decreased NF-κB p65 expression (Figure 2B).

### Effect of DHA on IκB-a degradation and NF-κB p65 nuclear localization

**Figure 3:**
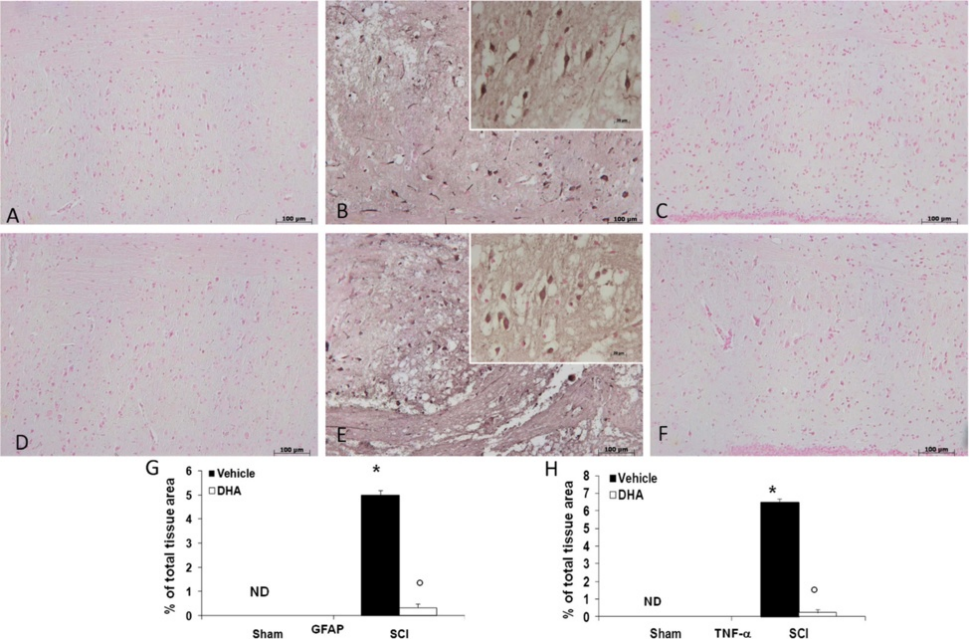
**Effects of DHA on astrocyte activation and TNF-α expression in spinal cord tissue.** Spinal cord sections were processed 24 h after SCI to determine the immunohistological staining for GFAP and TNF-α expression. Sham animals never express GFAP (**A**, see densitometry analysis **G**), the number of GFAP^+^ cells was significantly increased upon induction of SCI, due to astrocytes proliferation around the central canal (**B**, see densitometry analysis **G**).Treatment with DHA significantly decreased the activation of astrocyte-GFAP^+^ cells (**C**, see densitometry analysis **G**).Moreover, no positive staining for TNF-α was found in the spinal cord tissue from sham-operated mice (**D**, see densitometry analysis **H**). A substantial increase in TNF-α expression was found in spinal cord tissues from SCI mice 24 h after SCI (**E**, see densitometry analysis **H**). DHA treatment significantly attenuated TNF-α levels in the spinal cord (**F**, see densitometry analysis **H**). **(G,H)** Densitometry analysis of immunohistochemistry photographs (*n* = 5 photos from each sample collected from all mice in each experimental group) for GFAP and TNF-α from spinal cord tissues. The figure is representative of at least three experiments performed on different experimental days. For each SCI group see the high magnification of the images. Data are expressed as percentage of total tissue area. Data are mean ± SEM of ten mice for each group.*P < 0.05 vs. Sham. °*P* < 0.01 vs. SCI. ND: not detectable.

It has been known that after SCI, astrocytes become reactive as a response to damage [25]. In this study, using GFAP staining, we show that SCI induces a glial response, which reflects that astrocytes become activated, as indicated by staining for GFAP cells in injured animals compared with sham animals (Figures 3B and A respectively, see densitometric analysis, Figure 3G). Instead, the treatment with DHA decreased the number of reactive astrocytes (GFAP-activated astrocytes) (Figure 3C, see densitometric analysis Figure 3G).

To test whether DHA modulates the inflammatory process through the regulation of the secretion of pro-inflammatory cytokines, we analyzed the spinal cord levels of TNF-α, by immunohistochemical staining. There was no staining for TNF-α in spinal cord obtained from sham mice (Figure 3D, see densitometric analysis H). A substantial increase in TNF-α expression was found in inflammatory cells as well as in nuclei of Schwann cells in the white and gray matter of the spinal cord tissues collected from mice at 24 h after SCI (Figure 3E, see densitometric analysis Figure 3H). Spinal cord expression of TNF-α was significantly attenuated in DHA-treated SCI mice in comparison with SCI animals (Figure 3F, see densitometric analysis Figure 3H).

**Figure 4:**
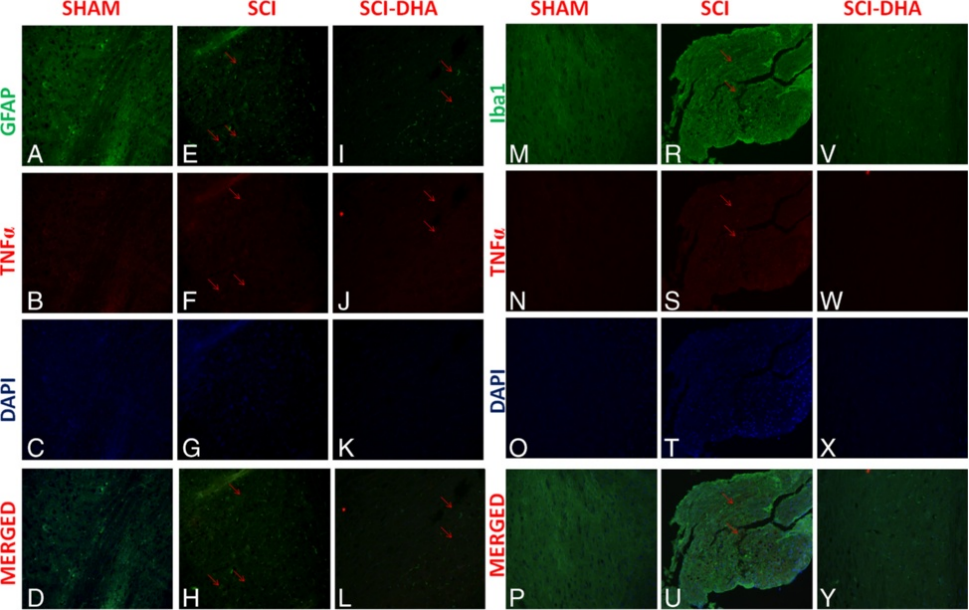
**Colocalization of GFAP/TNFα and Iba1/TNFα after SCI.** Results are shown for **(A-D, M-P)** sham-operated mice, **(E-H, R-U)** mice with SCI and **(I-L, V-Y)** mice with SCI treated with DHA. Spinal cord sections were double stained with antibodies against GFAP (**A**,**E**,**I**, green), Iba1 (**M**,**R**,**V**, green) and TNFα (**B**,**F**,**J**,**N**,**S**,**W**, red). Spinal cord sections revealed increased astrogliosis (GFAP + cells) in SCI mice **(E, R)**. GFAP immunoreactivity was reduced in DHA-treated mice **(I, V)**. Yellow spots indicate co-localizations **(H, U)** and revealed a high colocalization between GFAP/TNFα and Iba1/TNFα double staining.

Moreover, to confirm astrogliosis and TNFα expression and to localize it to specific cells types, we performed immunofluorescence staining. Spinal cord sections were double stained with antibodies against GFAP (green), Iba1 (green) and TNFα (red). Spinal cord sections revealed increased astrogliosis (GFAP + cells) in SCI mice (Figure 4E,H) such as activation of the microglia (Figure 4R). GFAP immunoreactivity and microglia activation were significantly reduced in SCI-treated mice (Figure 4I,L). The yellow spots indicate the colocalization between TNFα and GFAP (Figure 4H,L) a as well as between TNFα and Iba1 (Figure 4U,Y). Reported images are representative of triplicate experiments. All images were digitalized at a resolution of 8 bits into an array of 2048 × 2048 pixels.

### DHA modulates iNOS expression and nitrotyrosine formation after SCI

**Figure 5:**
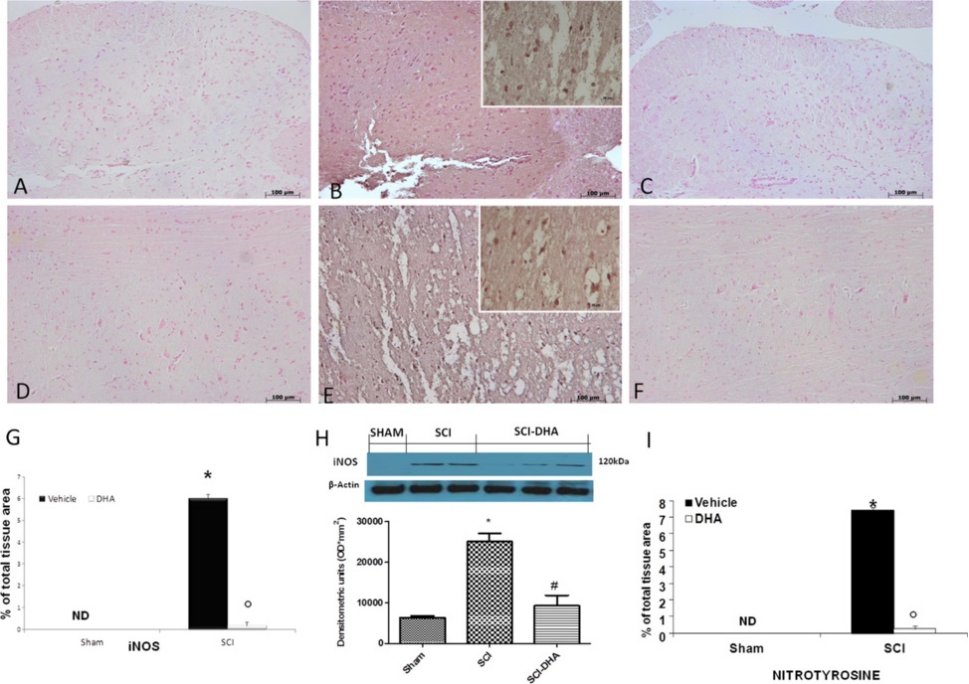
**Effects of DHA on nitrotyrosine formation and iNOS expression.** Immunohistochemical and Western blot analysis in the spinal cord section 24 h after SCI. Sections from sham-operated mice did not stain for iNOS (**A**, see densitometry analysis **G**), whereas those from SCI-operated mice exhibited staining for iNOS (**B**, see densitometry analysis **G**) mainly in various inflammatory cells in the gray matter. Treatment with DHA reduced the degree of staining for iNOS (**C**, see densitometry analysis **G**). Moreover, by Western blot analysis we observed high levels of iNOS in the SCI group **(H)**, whereas the DHA treatment group revealed a low expression of iNOS **(H)**. Thus, the tissue sections obtained from SCI mice demonstrate staining for nitrotyrosine mainly localized in inflammatory cells and in Schwann cell nuclei in the white and gray matter (**E**, see densitometry analysis **I**). DHA treatment reduced the degree of staining for nitrotyrosine (**F**, see densitometry analysis **I**). Conversely, spinal cord from sham-operated mice did not stain for nitrotyrosine (**D**, see densitometry analysis **I**). For each SCI group, see the high magnification of the images. **(G,I)** Densitometry analysis of immunocytochemistry photographs (*n* = 5 photos from each sample collected from all mice in each experimental group) for iNOS and nitrotyrosine. Data expressed as a percentage of total tissue area. This figure is representative of at least three experiments performed on different experimental days. Data are mean ± SEM of ten mice for each group.*P < 0.01 vs sham; °*P* < 0.01 vs SCI; ND: not detectable.

To determine the levels of NO produced during SCI, iNOS expression was evaluated by immunohistochemical analysis and Western blotting in the spinal cord sections 24 h after SCI. Spinal cord sections from sham-operated mice did not stain for iNOS (Figure 5A, see densitometric analysis Figure 5G), whereas spinal cord sections obtained from SCI-operated mice exhibited positive staining for iNOS (Figure 5B, see densitometric analysis Figure 5G), mainly localized in various inflammatory cells in the gray matter. Treatment with DHA reduced the degree of positive staining for iNOS in the spinal cord tissues (Figure 5C, see densitometric analysis Figure 5G). Moreover, by Western blot analysis we observed an increased expression of iNOS levels in the group subject to SCI compared with the control group (Figure 4H); while the treatment with DHA reduced iNOS expression significantly (Figure 5H).

Moreover, 24 h after SCI, nitrotyrosine, a specific marker of nitrosative stress, was measured by immunohistochemical analysis in the spinal cord sections to determine the localization of “peroxynitrite formation” and/or other nitrogen derivative produced during SCI. Spinal cord sections from sham-operated mice did not stain for nitrotyrosine, whereas spinal cord sections obtained from SCI mice exhibited positive staining for nitrotyrosine (Figure 5D and E respectively, see densitometric analysis I). The positive staining was mainly localized in inflammatory cells as well as in the nuclei of Schwann cells in the white and gray matter of the spinal cord tissues. DHA reduced the degree of positive staining for nitrotyrosine in the spinal cord (Figure 5F see densitometric analysis I, data are means ± SEM of 10 mice for each group *p < 0.01 vs. sham, °p < 0.01 vs SCI and vehicle. ND: not detectable).

### DHA modulates expression of Fas ligand after SCI

**Figure 6:**
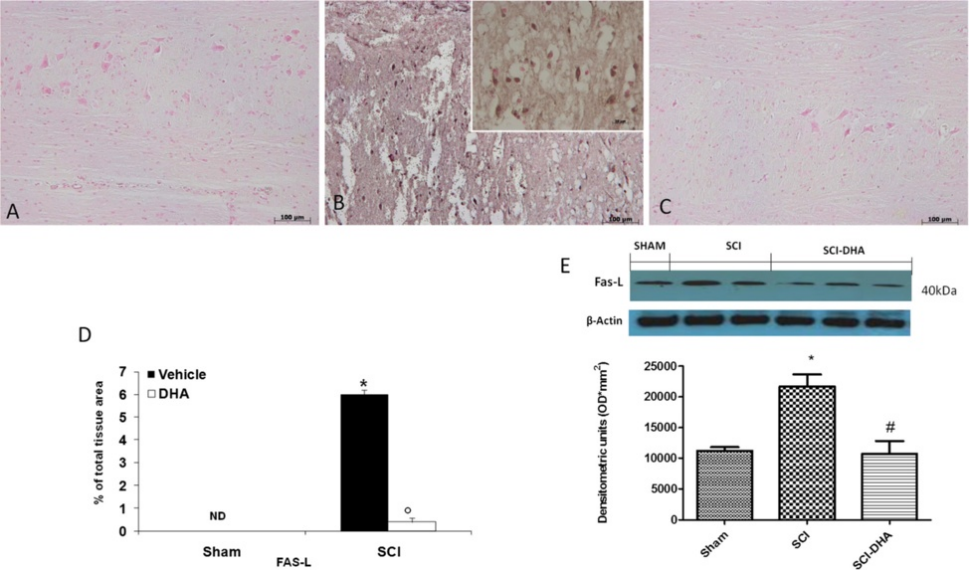
**DHA treatment reduced Fas-ligand expression in the perilesional spinal cord tissue.** No positive staining for Fas ligand was found in the spinal cord tissue collected from sham-operated mice (**A**, see densitometry analysis **D**). A substantial increase in Fas-ligand expression was found in inflammatory cells, and in white and gray matter of spinal cord tissues collected at 24 h after SCI (**B**, see densitometry analysis **D**). Spinal cord levels of Fas ligand were significantly attenuated by DHA treatment (**C**, see densitometry analysis **D**). **(D)** Densitometry analysis of immunocytochemistry photographs (*n* = 5 photos from each sample collected from all mice in each experimental group) for Fas ligand. Moreover Western blot analysis revealed an increased expression of Fas-L in the SCI group **(E)**, whereas DHA treatment significantly reduced the increased levels of Fas-L **(E)**. Figure is representative of at least three experiments performed on different experimental days. Data are mean ± SEM of ten mice for each group. *P < 0.01 vs Sham; °*P* < 0.01 vs SCI. # *P*<0.01 vs SCI; ND: not detectable.

Immunohistochemical staining for Fas ligand in the spinal cord was also determined at 24 h after injury. Spinal cord sections from sham-operated mice did not stain for Fas ligand, whereas those obtained from SCI mice exhibited positive staining for Fas ligand (Figure 6A, see densitometric analysis Figure 6D) mainly localized in various cells in the gray matter. Treatment with DHA reduced the degree of positive staining for Fas ligand in the spinal cord (Figure 6C, see densitometric analysis Figure 6D). Moreover, we Western blot analysis for Fas-L revealed an increased expression in the group subject to SCI (Figure 6E) compared with the control group, while treatment with DHA reduced Fas-L expression significantly (Figure 6E).

### Effects of DHA on apoptosis in spinal cord after injury

**Figure 7:**
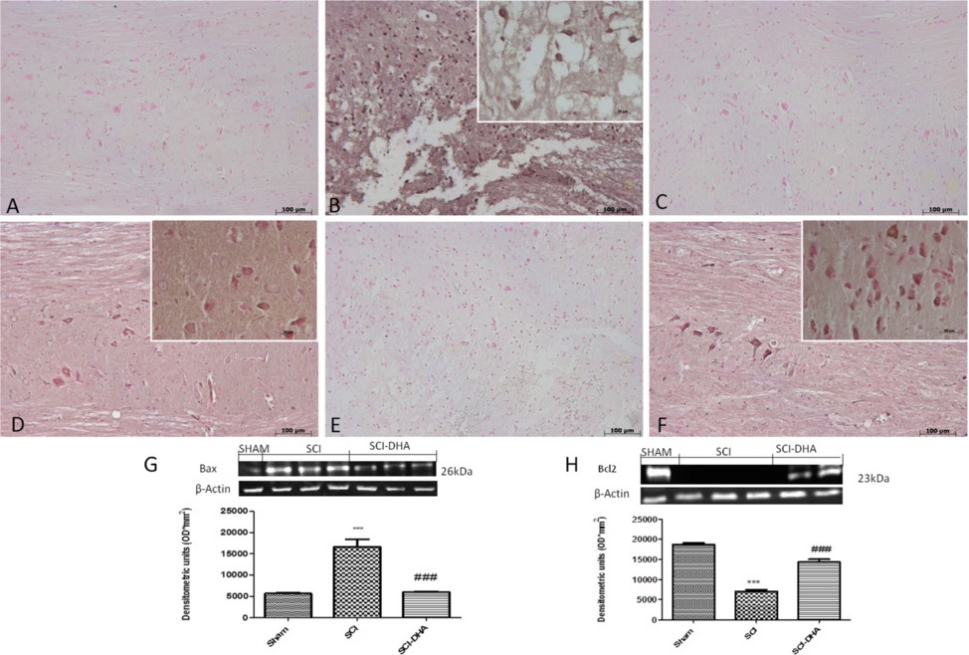
**Effects of DHA on intrinsic apoptotic pathway.** Staining for Bax expression was absent in the sham group **(A)**. Twenty-four hours after SCI, spinal cord tissue from injured animals showed positive staining for Bax **(B)**. DHA treatments significantly reduced the SCI-induced Bax **(C)**. On the contrary, positive staining for Bcl-2 was observed in the spinal cord tissues from sham-operated mice **(D)** while the staining was significantly reduced in SCI mice **(E)**. DHA treatment attenuated the loss of positive staining for Bcl-2 in the spinal cord from SCI- subjected mice **(F)**. For Bax staining in SCI groups, see the high magnification of the images; for Bcl2 staining, see high magnification for sham and DHA groups. Moreover, an increase in Bax expression was evidenced by Western blot analysis **(G)**. DHA treatment reduced the expression for Bax in the spinal cord ganglia). A basal level of Bcl-2 expression was detected in spinal cord from sham-operated mice **(H)**. Twenty-four hours after SCI, Bcl-2 expression was significantly reduced in spinal cord from SCI mice **(H)**. DHA treatments significantly reduced the SCI-induced inhibition of Bcl-2 expression **(H)**. A representative blot of lysates obtained from each group is shown, and densitometry analysis of all animals is reported. Respective densitometry analysis of protein bands from three separated experiments is reported. Data are mean ± SEM of ten mice for each group; ***P < 0.001 vs. sham; ^###^*P* < 0.005 vs. SCI. ND: not detectable.

To test whether spinal cord damage was associated with cell death, we evaluated the appearance of the pro-apoptotic protein Bax by Western blot analysis at 24 h after SCI. Bax levels were appreciably increased in the spinal cord tissue collected from mice subjected to SCI (Figure 7G). In contrast, treatment with DHA prevented SCI-induced Bax expression (Figure 7G). To detect Bcl-2 expression, whole extracts from the spinal cord of each mouse were also analyzed by Western blotting. Expression of the anti-apoptotic protein Bcl-2 was apparent in spinal cord homogenates from sham-operated mice (Figure 7H). Twenty-four hours after SCI, Bcl-2 expression was significantly reduced in spinal cord samples from SCI mice (Figure 7H). Treatment of mice with DHA significantly blunted the SCI-induced inhibition of Bcl-2 expression (Figure 7H).

To confirm our data on apoptotic proteins, we also determined Bax and Bcl-2 expression by immunohistochemical staining. Immunohistochemical staining for Bax and Bcl-2 also showed that spinal cord sections from sham-operated mice did not stain for Bax (Figure 7A), whereas SCI-operated mice exhibited positive staining for Bax (Figure 7B). DHA treatment reduced the degree of positive staining for Bax in spinal cord samples from mice subjected to SCI (Figure 7C).

In addition, spinal cord sections from sham-operated mice demonstrated Bcl-2 positive staining (Figure 7D), whereas in SCI mice, the staining was significantly reduced (Figure 7E). Treatment with DHA attenuated the loss of positive staining for Bcl-2 in the spinal cord from SCI-subjected mice (Figure 7F).

**Figure 8:**
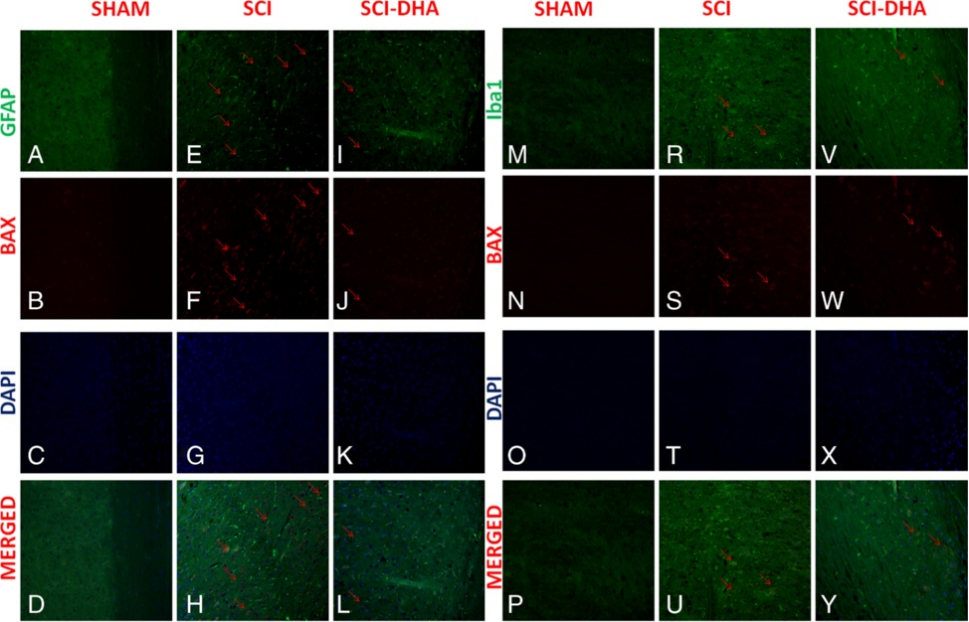
**Colocalization of GFAP/Bax and Iba1/Bax after SCI.** Results are shown for **(A-D, M-P)** sham-operated mice, **(E-H, R-U)** mice with SCI and **(I-L, V-Y)** mice with SCI treated with DHA. Spinal cord sections were double stained with antibodies against GFAP (**A,E,I,** green), Iba1 (**M,R,V,** green) and Bax (**B,F,J,N,S,W**, red). Spinal cord sections revealed increased Bax expression in SCI mice **(F,S)**. Bax expression was reduced in DHA-treated mice **(J,W)**. Yellow spots indicate co-localizations **(H,U)** and revealed a high colocalization between GFAP/Bax and Iba1/Bax double staining.

Moreover, to confirm Bax expression and to localize it to specific cell types, we performed immunofluorescence staining. Spinal cord sections were double stained with antibodies against GFAP (green), Iba1 (green) and Bax (red). Spinal cord sections revealed increased expression of Bax in SCI mice (Figure 8F,S). Bax activation was reduced in SCI-treated mice (Figure 8J,W). The yellow spots indicate the colocalization between Bax and GFAP (Figure 8H,L) as well as between Bax and Iba1 (Figure 8U,Y). Reported images are representative of triplicate experiments. All images were digitalized at a resolution of 8 bits into an array of 2048 × 2048 pixels.

### Role of functional PPAR-α gene in the protective and anti-inflammatory properties of DHA of the degree of spinal cord trauma

**Figure 9:**
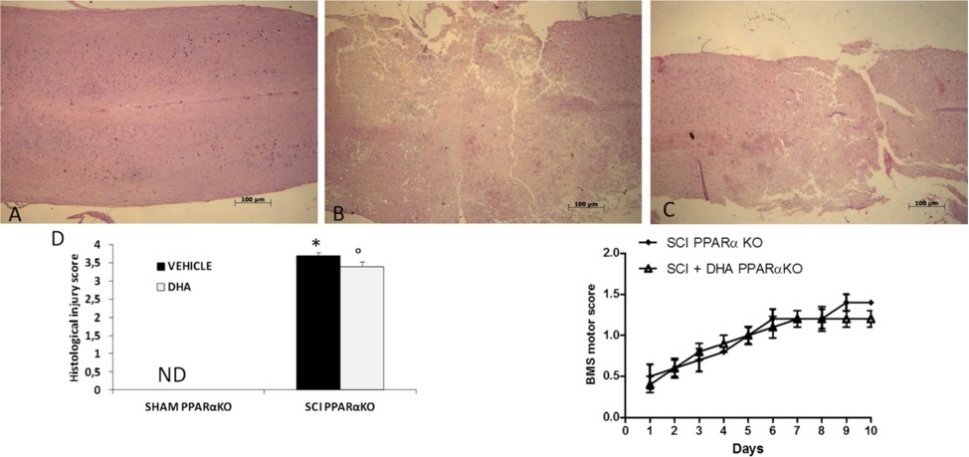
**Involvement of peroxisome proliferator-activated receptors on the protective actions of DHA following spinal cord trauma.** Following spinal cord compression, significant damage to the spinal cord from PPARα KO mice **(B)** at the perilesional zone was observed by H & E staining when compared with spinal cord tissue collected from the sham group **(A)**. The genetic absence of the PPAR-α receptor significantly blocked the effect of the DHA treatment **(C)**. The histological score **(D)** was made by an independent observer. These figures are representative of at least three experiments performed on different experimental days. Data are mean ± SEM of ten mice for each group. *P < 0.01 vs sham; °*P* < 0.01 vs SCI.

As PPAR-α is constitutively expressed in astrocytes and neurons, it has been proposed that PPAR-α regulates brain and spinal cord lipid homeostasis during physiological conditions. PPAR-α activation may improve fatty acid mobilization, supporting both functional modification and structural reorganization in the dorsal horn of the spinal cord, such as activation of the arachidonate cascade, or axon sprouting. To better study the mechanism of action of DHA, we inflicted SCI on PPARα KO mice. Spinal cords from uninjured mice appeared normal by gross and microscopic examination (Figure 9A, see histological score Figure 9D). Twenty-four hours after the trauma, intraparenchymal hemorrhages and cell swelling were visible throughout the white matter of injured cords (Figure 9B, see histological score Figure 9D) compared with sham-operated mice. PPAR-α deletion increased spinal cord edema, which is a well-recognized cause of secondary neuronal damage after SCI in human beings and animals. The genetic absence of the PPAR-α in KO mice significantly reduced the effect of the DHA treatment (Figure 9C, see histological score Figure 9D).

Traumatic SCI results in severe inflammation and decreased cellular regeneration, which lead to difficult functional recovery. Open-field locomotor function was tested using the BMS, from 1 day post injury through to the end of the study (10 days). While motor function was only slightly impaired in sham-operated mice, a significant increase in hind limb motor disturbances was observed in the PPAR-αKO operated mice (Figure 9E). The genetic absence of the PPAR-α receptor significantly blocked the effect of the DHA treatment (Figure 9E).

### *In-vitro* study

#### ***Effect of DHA on neurite growth***

In the DRG culture, approximately 15% of the total cells are neuronal (β-tubulin III positive) and 20% are Schwann cells. The remainder of the non-neuronal cells are mainly satellite cells, with some fibroblasts. Each DRG subpopulation was determined as a percentage of the total neuron population, identified by counterstaining with the pan-neuronal marker β-tubulin III (mouse).

**Figure 10:**
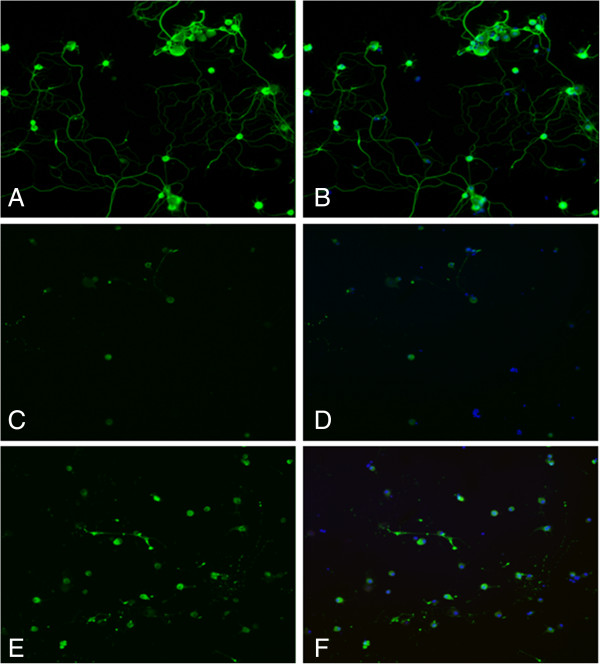
**Representative fluorescence microscopy images of DRG cultures stained with neuronal marker.** DRG cells were stained with β-tubulin III to visualize the neurites. Oxidative stress induced by the administration of H_2_O_2_ significantly decreased the outgrowth of the DRG neurons in culture **(C, D)** compared with the control DRG cells group **(A, B)**, whereas the treatment with DHA increased the percentage of cells with complex growth, similar to the control DRG cells **(E, F)**. Cells were counterstained with Hoechst.

To assess the morphology of the cells in more detail, DRG cells were stained with β-tubulin III for visualization of the neurites. As shown in Figure 10, the oxidative stress induced by the administration of H_2_O_2_ significantly decreased the outgrowth of the DRG neurons in culture (Figure 10C,D) compared with the control DRG cells group (Figure 10A,B), whereas treatment with DHA (1 μM) increased the percentage of cells with complex growth, similar to the control DRG cells (Figure 10E,F).

### Effect of DHA on neurite length and number of branches

**Figure 11:**
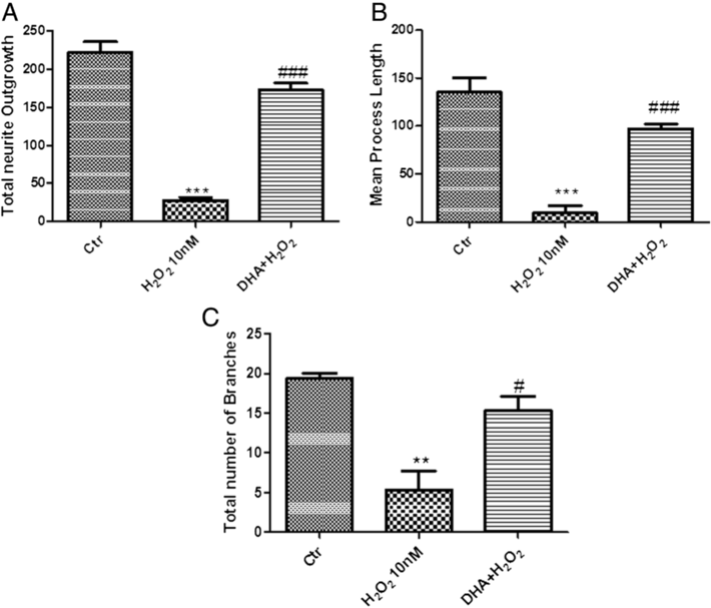
**Effect of DHA on neurite outgrowth.** Neurite process length and number of branches were significantly reduced in H_2_O_2_-injured cultures **(A,B,C)**, compared with control DRG cells **(A,B,C)**. Total outgrowth, length of longest neurite and number of branches were all markedly increased in the DRG cells treated with DHA **(A,B,C)**. Data are mean ± SEM of ten mice for each group. ***P < 0.001 vs Ctr; ### *P* < 0.005 vs H_2_O_2_; ** *P* < 0.01 vs Ctr; # *P* < 0,05 vs H_2_O_2_.

The number of neurons with neurites (as total outgrowth), the neurite process length, and the number of branches were all seen to be reduced in H_2_O_2_ injured cultures (Figure 11A,B,C, respectively), compared with control DRG cells group (Figure 11A,B,C, respectively). Injury may result in neurons halting their neurite growth, or may lead to a selective loss of cells that have long neurites. Total outgrowth, length of longest neurite and number of branches were all markedly increased in the DRG cells treated with DHA 1 μM (Figure 11A,B,C, respectively).

## Discussion

Spinal cord injury has a high prevalence and a profound impact on both the patient and on society as a whole. Functional recovery following SCI is often poor and, as yet, there are no nonsurgical therapies that have been translated to the clinic. The corticosteroid methylprednisolone is the only recognized pharmacological treatment that modulates the early inflammatory reaction after SCI in human beings [26]. However, methylprednisolone has not been included in the standard care of SCI patients worldwide, since its use has been shown to have adverse side effects, including metabolic complications and an increased risk of wound infection. Drug administration that is safe and efficacious is vital for optimal treatment. In the search for potential therapeutic targets, increasing attention has been focused on the molecular events that occur immediately after SCI. Direct mechanical damage to neurons, axons, glia cells and blood vessels induces the release of molecules that lead to glial activation and initiation of secondary inflammation. Various strategies to interfere with these events have been tested experimentally and some have been pursued in human clinical trials. However, the question of how to treat patients at this time still remains open. In this work, an *in-vivo* injury model of SCI was used, to replicate some of the typical features of the inflammatory conditions after SCI. Moreover, we tried to elucidate whether the exogenous administration of DHA can protect against SCI. Lang-Lazdunski *et al*. [22] had initially shown, in a model of transient spinal cord ischemia in rats, that the administration of an omega-3 fatty acid, α-linolenic acid, after injury, decreased neuronal loss and improved functional outcome. Moreover, previous studies of Lim *et al*. [16] demonstrated that omega-3 DHA, administered as an intravenous bolus after SCI induced by hemisection of the spinal cord in adult rats, induced significant neuroprotection, reducing neuronal cell loss and oligodendrocyte loss, and decreasing the apoptosis measured 1 or 6 weeks after injury. In addition, it has been shown that DHA can counteract glutamate-induced excitotoxicity. As inflammation, excitotoxicity and oxidative stress are part of the pathogenetic mechanism involved in the secondary injury associated with SCI [20], this led us to explore the potential of DHA as a neuroprotective agent, targeting the very early events after SCI, within 24 h. Spinal cord injury results in a necrotic area of cavitation that progressively increases in area due to secondary neuronal death promoted by acute inflammation, edema, apoptosis, and glial scarring. The acute loss of motor neurons and degeneration of white matter tracts results most often in irreversible motor dysfunction. In this work, we clearly demonstrated that DHA treatment attenuates the degree of secondary inflammation and improves motor recovery. This is consistent with previous results obtained in static compression SCI in rats [13,14] and mice [16]. A variety of experimental evidence suggests that activation of the transcription factor NF-κB plays a central role in the regulation of many genes responsible for the generation of mediators or proteins involved in secondary inflammation associated with SCI [27]. There is good evidence that ω-3 PUFAs inhibit the activation of NF-κB, by preventing the phosphorylation of IK kinase, hence preventing the degradation of IκB, and resulting in transactivation of NF-κB target genes [28]. DHA antagonizes the NF-κB signaling pathway, inhibiting the expression of inflammatory genes downstream of NF-κB. We report here that SCI is associated with significant IκB-α degradation as well as increased nuclear expression of p65 in spinal cord tissues 24 h after injury. Treatment with DHA significantly reduced IκB-α degradation as well as NF-κB localization. NF-κB plays a central role in the regulation of many genes responsible for the generation of mediators or proteins in inflammation. These include the genes for TNF-α, IL-1β, iNOS, and COX-2 [29]. In this regard, it has been well demonstrated that in SCI the expression of pro-inflammatory cytokines, such as TNF-α and IL-1β, regulates the precise cellular events after SCI. We confirm in this study that SCI leads to a substantial increase in the levels of TNF-α. Remarkably, there was no increase in the expression of TNF-α in the spinal cord sections obtained from mice treated with DHA. Therefore, the inhibition of the production of TNF-α by DHA described in this study can most probably be attributed to the inhibitory effect of the activation of NF-κB. Moreover, previous studies have shown that TNF-α activates iNOS and induces the generation of NO-radical (NO) from L-arginine [30]. This study demonstrates that DHA attenuates the expression of iNOS in the tissue from SCI-treated mice when compared with untreated injured mice. It has also been demonstrated that peroxynitrite, a cytotoxic molecule generated when nitric oxide (NO) and superoxide (O2^-^) combine, probably contributes to secondary neuronal damage through pathways resulting from the chemical modification of cellular proteins and lipids in the secondary neuronal damage of SCI [31]. Nitrotyrosine formation, along with its detection by immunostaining, was initially proposed as a relatively specific marker for the detection of the endogenous formation ‘footprint’ of peroxynitrite. Increased nitrotyrosine staining is considered, therefore, as a marker of increased nitrosative stress rather than a specific marker of the peroxynitrite generation. In this study, we have found that the damage induced by SCI is associated with an intense immunostaining for nitrotyrosine. We clearly demonstrated that treatment with DHA significantly prevents the appearance of nitrotyrosine staining.

Another important mediator of secondary damage after SCI is the apoptosis process [30]. We report in this study that the treatment with DHA in our SCI experimental model reduces apoptosis markers, suggesting that DHA could be beneficial for treatment of SCI. In particular, we demonstrate that the treatment with DHA reduced Bax expression while, conversely, Bcl-2 is expressed much more in mice treated with DHA. It can be hypothesized that DHA is able to attenuate this apoptotic cell death, which often occurs at a slower rate than necrosis, upregulating the expression of the Bcl-2 family of anti-apoptotic proteins, while downregulating the apoptotic proteases caspase-3 and 9, and pro-apoptotic signaling proteins including Bax, Bad, Bid, and Bik, in a number of cell types including neurons and oligodendrocytes. This could account for some of the neuroprotection seen in the present study.

In addition, a variety of evidence has shown that Fas ligand plays a central role in apoptosis induced by a variety of chemical and physical insults. Fas co-localizes in many TUNEL-positive cells, suggesting that Fas-initiated cell death cascades may participate in the demise of some glia following SCI. We clearly demonstrated that DHA significantly reduced the Fas-ligand expression induced by SCI. Based on this evidence, we have shown clearly that DHA interferes with the apoptotic process induced by SCI. However, it is not possible to exclude the possibility that the anti-apoptotic effects observed after DHA treatment are secondary to an attenuation of the inflammatory reaction.

Moreover, recent studies suggested that PUFAs, and in particular omega-3 fatty acids, are endogenous ligand for RXR and PPAR receptors. Thus, we hypothesized that a marked neuroprotective role of omega-3 PUFA is possibly driven by the activation of these receptors. So our study investigated whether the protective and anti-inflammatory effects of DHA observed in a compression model of SCI are partially mediated by PPAR receptor activation and in particular by PPAR-α isotypes. To characterize the role of PPAR-α in DHA-mediated anti-inflammatory and neuroprotective activities, we inflicted spinal cord trauma on PPARα KO mice. The results of this study indicated that PPAR-αKO mice are more susceptible to induction of SCI than are WT mice, possibly owing to a less efficient anti-inflammatory control exerted by endogenous PPAR-α ligand; moreover we demonstrated in this study that the genetic absence of the PPAR-α in KO mice significantly reduced the protective effect of the DHA treatment that we observed in WT mice. Results discussed here suggest a new mechanism of action for DHA contributing to determining the full DHA efficacy and suggest future studies should aim to analyze the possible relevance of the other PPAR isoforms, such as PPAR-β/δ and PPAR-γ, in DHA-mediated protection in SCI.

In this study, we also tested the efficacy of ω-3 PUFA in an *in-vitro* model of DRG cell culture [32]. In particular, we studied the role of DHA in an oxidative stress model induced by administration of H_2_O_2_ in culture medium in the presence or absence of DHA. Oxidative stress is associated with many CNS and peripheral nervous system (PNS) pathologies, including SCI, and protection of neuronal and non-neuronal cells against this type of injury is of considerable importance. The ability to protect neurons against cell death is crucial to successful recovery after a traumatic neuronal injury, since regeneration in the PNS can only occur from neurons that survive the initial trauma. In the case of trauma to the CNS, where spontaneous regeneration is severely limited, protection of neurons becomes even more critical. In this study, the *in-vitro* model of DRG cell culture demonstrated that the injury induced by H_2_O_2_ mediated oxidative stress was significantly attenuated by supplementation of the media with DHA (1 μM). Total outgrowth, length of longest neurite and number of branches were all increased in the cultures that received the administration of DHA (1 μM) compared to cultures that were subjected to the oxidative stress and had no treatment. The increase in neurite extension is in agreement with the work of others, which shows that a priming injury results in a rapidly elongating mode of growth of neurons *in vitro*[32].

This is the first study to demonstrate that DHA is neuroprotective in an *in-vitro* model of oxidative stress in DRG neurons. The *in-vitro* model allowed for an investigation into the neuroprotective effects of ω-3 PUFAs in adult DRG cultures. DHA was found to be a very potent neuroprotector, and this is one of the key findings of the work. This study also confirmed that DHA was neuroprotective when given as a post-injury treatment. This suggests that DHA could be of benefit when administered after a traumatic injury, which is an important factor when considering translation to the clinic.

## Conclusions

*In-vitro* models are a useful tool for screening neuroprotective agents and for determining their mechanisms of action. Nevertheless, testing *in vivo* is essential for the development of a therapy and successful translation from the bench to the bedside. The results obtained in this work also provide an indication that DHA could increase the rate of regeneration of neurons and axons after trauma. To conclude, both *in-vitro* and *in-vivo* experiments point to the neuroprotective and neurotrophic effects of long chain ω-3 PUFAs, such as DHA, which could also lead to a secondary effect on regeneration following a SCI. This potential avenue demonstrates that further research into the mechanisms leading to the increased rate of recovery after DHA is warranted.

## Abbreviations

ANOVA: Analysis of variance; BMS: Basso mouse scale; BSA: Bovine serum albumin; CNS: Central nervous system; EPA: Eicosapentaenoic acid; DHA: Docosahexaenoic acid; DRG: Dorsal root ganglia; Fas-L: Fas ligand; GFAP: Glial fibrillary acidic protein; H & E: Hematoxylin and eosin; IgG: Immunoglobulin G; iNOS: Inducible nitric oxide synthase; NF: Nuclear factor; PAGE: Polyacrylamide gel electrophoresis; PBS: Phosphate buffered saline; PMSF: Phenylmethylsulfonyl fluoride; PUFA: Polyunsaturated fatty acid; SCI: Spinal cord injury; SEM: Standard error of the mean; TNF: Tumor necrosis factor; WT: Wild-type.

## Competing interest

The author(s) declare that they have no competing interests.

## Authors’ contributions

IP and EE performed experiments and prepared the manuscript. DI and RDP performed *in-vivo* experiments and the biochemical analysis. SG and PY performed *in-vitro* experiments. JVP, ATM-T, and SC planned the study, analyzed the results, and prepared the manuscript. All authors read and approved the final manuscript.
